# Ethnic diversity in precision medicine: a reality or an aspiration?

**DOI:** 10.1007/s00125-025-06513-4

**Published:** 2025-08-07

**Authors:** Shivani Misra

**Affiliations:** 1 Division of Metabolism, Digestion and Reproduction, Imperial College London, London, UK; 2 Department of Diabetes & Endocrinology, Imperial College Healthcare NHS Trust, London, UK

**Keywords:** Ancestry, Equity, diversity and inclusion, Ethnicity, Heterogeneity, Personalised medicine, Precision medicine, Review, Type 1 diabetes, Type 2 diabetes

## Abstract

Precision medicine seeks to tailor prevention, diagnosis and treatment strategies to the biological and contextual characteristics of individuals. In diabetes, where there is substantial heterogeneity in risk, progression and treatment response, this approach holds particular promise. However, the majority of precision medicine research has been conducted in populations of White European ancestry, limiting its relevance and equity across ethnically diverse groups. This review examines the role of ethnicity in precision diabetes medicine, highlighting its potential value and limitations. It summarises evidence for ethnic variation in both type 1 and type 2 diabetes, including differences in susceptibility, phenotypes and complications, and demonstrates how under-representation in research has led to diagnostic and therapeutic blind spots. Three conceptual models for incorporating ethnicity into precision approaches are described: ancestry-anchored discovery, ethnicity-adapted translation and trait-based individualisation. Each model presents distinct opportunities for and challenges to equitable implementation. The review also outlines key barriers, such as limited diversity in discovery science, inconsistent data infrastructure, and resource constraints, and proposes practical strategies to overcome them. It concludes by identifying critical research gaps and emphasises the need for inclusive and context-sensitive models of precision medicine that account for both inter- and intra-ethnic diversity. Realising this vision will be essential to delivering effective and equitable diabetes care globally.

**Graphical Abstract:**

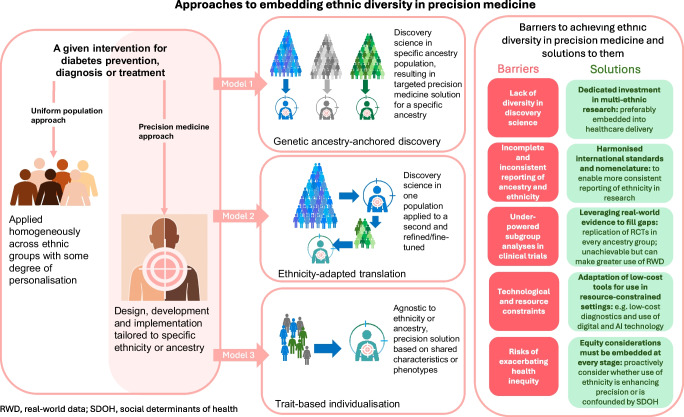

**Supplementary Information:**

The online version contains a slideset of the figures for download available at 10.1007/s00125-025-06513-4.

## Introduction

The concept of precision medicine has rapidly evolved from a theoretical approach to a practical goal across many areas of clinical care [1]. Precision medicine represents a shift from uniform, population-level treatment strategies towards individualised care tailored to a person’s unique biological, phenotypic and sociocultural profile. In diabetes, where there is considerable heterogeneity in pathophysiology, clinical course and treatment response, the need for such a paradigm shift is arguably urgent. While traditional models of care have focused on glycaemic thresholds and broad therapeutic algorithms, precision medicine has the potential to improve diagnostic accuracy, therapeutic efficacy and long-term outcomes by accounting for variability across individuals and populations [2, 3]; the case for moving beyond ‘one-size-fits-all’ models is therefore particularly compelling. Advances in molecular diagnostics, clinical informatics and data science have enabled increasingly sophisticated approaches to stratifying patients by risk and guiding individualised treatment [4]; however, the translation of these innovations into clinical practice with equitable reach remains elusive.

For precision medicine to be truly transformative, it must also be equitable. This requires confronting the historical under-representation of ethnically diverse populations in diabetes research and recognising the complex ways in which race, ethnicity and ancestry intersect with disease risk and clinical decision-making. Ethnicity has long been acknowledged as a modifier of diabetes risk and phenotype. Epidemiological studies consistently demonstrate a higher prevalence of type 2 diabetes in populations of South Asian, East Asian, African, Middle Eastern and Hispanic descent [5], often with earlier onset [6], a leaner body composition [7] and differences in beta cell function [8] and fat distribution [9], underscoring the inadequacy of one-size-fits-all approaches to prevention and treatment.

Despite these well-recognised differences, many tools used in precision diabetes medicine to date, including genetic risk scores, clustering algorithms and prediction tools, have been developed and validated in predominantly White European populations [10]. This lack of diversity limits generalisability and may entrench existing inequities if tools are applied uncritically to these under-represented groups.

While commonly used in biomedical research, ethnicity is a socially defined construct that does not map directly onto genetic ancestry and may instead reflect a mixture of sociocultural, behavioural, environmental and systemic exposures [11]. In some contexts, the use of race or ethnicity in clinical algorithms, such as in adjustments to eGFR or risk prediction scores, has reinforced structural biases, delaying diagnosis and compromising care [12–14].

This paradox of ethnicity as both a potential tool for enhancing precision and a potential source of inequity sits at the heart of this review. The challenge is to develop approaches that recognise meaningful variation between and within ethnic groups, while avoiding reductionist models that overlook structural determinants of health.

This review explores the evidence for ethnic variation in diabetes risk, presentation and outcomes; the strengths and limitations of incorporating ethnicity into clinical and research frameworks; and conceptual models for integrating ethnic diversity into precision approaches. Finally, the barriers to equitable implementation, including data gaps, are highlighted, and pragmatic solutions to advance inclusive, scalable and ethically grounded precision diabetes care are proposed.

## Ethnicity, race and ancestry

Race, ethnicity and ancestry are distinct but overlapping constructs used in biomedical research but are often used interchangeably [15]. Race refers to a grouping based on physical characteristics and is widely recognised as a social rather than a biological categorisation that does not reflect clearly defined genetic boundaries [16]. Ethnicity encompasses shared cultural, linguistic or ancestral traits and may include factors such as nationality, religion and geographical origin [11]. Although neither race nor ethnicity serves as a precise proxy for genetic variation, both remain relevant in epidemiological studies because of their association with differences in health behaviours, environmental exposures, healthcare access and broader social determinants of health [17, 18]. Ancestry specifically refers to patterns of genetic inheritance and is usually studied through genetic markers that reflect the geographical origins of an individual’s ancestors [19]. Ancestry can provide insights into genomic variation relevant to disease risk or drug response, particularly in studies where self-identified race or ethnicity may not align with genetic background. Genetic ancestry therefore reflects biological lineage, while race and ethnicity are shaped by social, political and cultural contexts [20].

A fundamental question is whether using ethnicity or race as a modifier of clinical decision-making has value or whether, as some suggest, it worsens health inequalities [20]. A recent example is the adjustment for Black ethnicity in equations used for estimating eGFR. The original equation was based on data from only 195 African American individuals and suggested, without confirmed causal evidence, that higher serum creatinine levels reflected greater muscle mass. This led to a 16% upward correction in eGFR for individuals classified as Black [12]. However, subsequent data suggest that this adjustment misclassified chronic kidney disease in up to 24% of Black individuals, potentially delaying referrals, transplant listing and appropriate surveillance [21].

Increasing awareness of how socioeconomic and ethnic factors contribute to global health disparities has led to growing criticism of using race or ethnicity as adjustment variables in clinical algorithms [12, 20]. As race and ethnicity are socially defined rather than biologically determined, using them as proxies for physiological variation is problematic, as differences may arise from the interaction of genetic, environmental and socioeconomic influences [22]. Similarly, the binary classification of populations, such as ‘White’ vs ‘non-White’, risks oversimplification and obscures the substantial diversity that exists within ethnicities. On the other hand, not taking ethnicity into consideration overlooks an important characteristic that can significantly influence disease risk and outcomes, particularly in conditions such as diabetes. The solution is likely to be a transition from using broad, socially constructed categories such as race or ethnicity towards more precise approaches that integrate genetic ancestry, molecular profiling and individual-level data on environment and lifestyle. This shift would lay the groundwork for more targeted and equitable strategies in clinical care and aligns with the goals of precision medicine.

## What is precision medicine for diabetes?

Precision medicine is an end-to-end clinical approach that emphasises the tailoring of prevention efforts, diagnostics and/or therapeutics to subgroups of populations sharing similar characteristics [1]. In traditional evidence-based medicine, interventions are typically applied homogeneously across a population, with little or no adjustment for the characteristics of an individual, with the rationale being that small improvements across the entire population are more beneficial than large improvements in small segments of the population [23].

The second international consensus statement on precision medicine for diabetes highlighted that adopting a precision medicine approach had the potential to minimise diagnostic and therapeutic errors and improve the accuracy, safety, cost-effectiveness, accessibility and individual relevance of medical decisions and health recommendations [1].

While precision medicine has often been associated with expensive ‘omics technologies yielding incremental benefits, there is a growing consensus that it more broadly refers to tailoring care based on clinical characteristics, biomarkers or molecular profiling. In practice, many clinicians already engage in informal, experience-based personalisation, adjusting treatment according to individuals' socioeconomic context, family history, lifestyle and comorbidities. These longstanding practices can be seen as a form of intuitive precision medicine.

At its simplest, ethnicity may be considered a proxy for a subgroup of individuals sharing certain phenotypic or sociocultural characteristics. With this perspective, it is plausible that precision medicine could provide advantages over empirical models of care, typically derived from studies in majority White European populations.

## Ethnic variation in type 2 diabetes

**Fig. 1:**
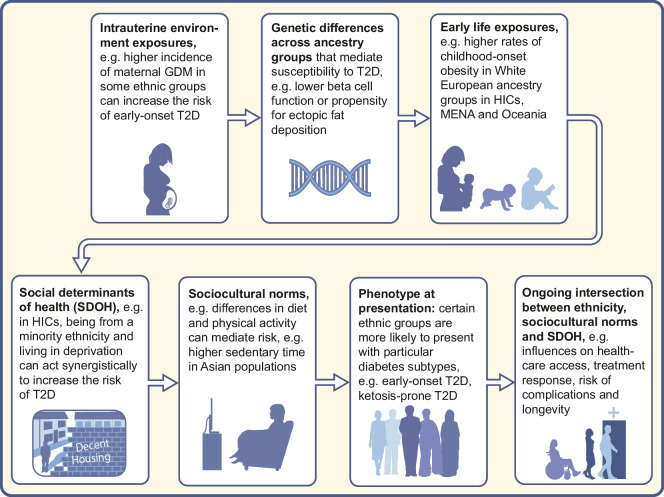
Schematic diagram illustrating how ethnicity can impact type 2 diabetes risk and outcomes across the life course. GDM, gestational diabetes; MENA, Middle Eastern and North Africa; T2D, type 2 diabetes. This figure is available as part of a downloadable slideset

Incorporating ethnicity into diabetes research and care is grounded in the recognition that differences in diabetes risk, its pathophysiology and treatment response are observed across ethnic groups. A uniform approach to all ethnic groups may overlook important variation, potentially limiting both the effectiveness and the equity of interventions. This principle extends beyond ethnicity to other under-represented groups, such as women [24] and younger individuals with diabetes [25], who are under-represented in clinical trials in diabetes. As a result, guidelines and risk assessment tools may not reflect the clinical realities of diverse populations. Ethnicity is likely to impact type 2 diabetes across the life course (Fig. 1) and the evidence for ethnic variation in type 2 diabetes is reviewed below.

### Type 2 diabetes risk

The impact of ethnicity in modulating phenotype has been most extensively studied in type 2 diabetes. Today, approximately four out of five people living with type 2 diabetes inhabit low- and middle-income countries (LMICs) [26]. Individuals of South Asian, East Asian, African, Middle Eastern and North African (MENA) and Hispanic ethnicities exhibit significantly higher risks of developing type 2 diabetes than individuals of White European ethnicity. The highest age-standardised prevalence of type 2 diabetes is reported in MENA populations (19.9%), followed by North American (13.8%), East Asian (11.1%) and South Asian (10.8%) populations; this compares with prevalences of 8% in Europe and 5% in Africa. Notably, the largest projected increases in type 2 diabetes prevalence over the next 25 years are expected in Africa, MENA regions and South Asia [26]. Within-country comparisons further highlight the differential risk. The SABRE study, examining UK-based migrant populations, reported age-adjusted HRs for incident type 2 diabetes of 2.88 (95% CI 2.36, 3.53) for Indian Asian men and 2.23 (1.64, 3.03) for African Caribbean men compared with White British men, with similarly elevated risks among Indian Asian and African Caribbean women [27]. In women with a history of gestational diabetes, the cumulative incidence of type 2 diabetes over follow-up is substantially higher in non-White European ethnicities [28]. Indeed, the risk of developing gestational diabetes is also much greater in Asian and Black pregnant women than in those of White European ethnicity [29].

These epidemiological differences prompt important questions about whether traditional risk factors, particularly excess adiposity, mediate disease uniformly across ethnic groups. Ethnic-specific differences in genetic susceptibility are likely to modify the impact of adiposity on type 2 diabetes risk [9, 30, 31]. South Asian and East Asian populations, for instance, experience significantly greater type 2 diabetes risk at lower BMI [27, 32–34]. Recognising this, the ADA recommends earlier screening for type 2 diabetes in Asian American adults with a BMI ≥23 kg/m^2^ [35] and other guidelines define overweight in Asian and Black ethnicity groups as a BMI >23.5 kg/m^2^ [32]. Applying uniform BMI thresholds could therefore delay diagnosis and undermine prevention efforts in these high-risk ethnic groups.

One proposed mechanism for the heightened susceptibility to type 2 diabetes among certain populations is a greater propensity for ectopic fat accumulation, particularly hepatic and visceral fat [36, 37]. Mendelian randomisation studies have demonstrated that ectopic fat deposition causally contributes to insulin resistance, independent of overall adiposity [38]. Partitioned polygenic scores (genetic risk scores for particular phenotypic traits) for lipodystrophy-related risk also suggest a higher burden of genetic risk for adverse fat distribution abnormalities among East Asian individuals. In one large multi-ancestry study, the equivalent BMI associated with type 2 diabetes risk was 30 kg/m^2^ in European populations but only 24.2 kg/m^2^ in East Asian populations; however, after adjusting for lipodystrophy-specific polygenic risk, this threshold in East Asian populations increased to 28.5 kg/m^2^, highlighting the significant contribution of genetic factors [9].

Beyond adiposity, differences in beta cell function and insulin secretory capacity appear central to ethnic differences in diabetes risk. Several studies report lower partitioned polygenic scores for beta cell function among South Asian and East Asian individuals with type 2 diabetes, predisposing these populations to an earlier age of onset of type 2 diabetes and more severe beta cell failure [8, 39, 40]. Lower lean muscle mass, a key determinant of insulin sensitivity, has also been observed across Asian ethnic groups and may amplify vulnerability to type 2 diabetes risk, as skeletal muscle mass independently mediates glucose clearance [41–44].

Maternal undernutrition, infection and obesity and gestational diabetes can impair fetal growth, reducing metabolic resilience and beta cell capacity from birth. These factors, particularly prevalent in LMIC settings, interact with rapid urbanisation, obesogenic environments and sedentary lifestyles to amplify diabetes risk in adulthood [45]. Taken together, there is substantial evidence to support differential risk of type 2 diabetes across different ethnic groups, shaped in part by genetic predisposition but also by environmental exposures (Fig. 1).

### Type 2 diabetes phenotypes

**Fig. 2:**
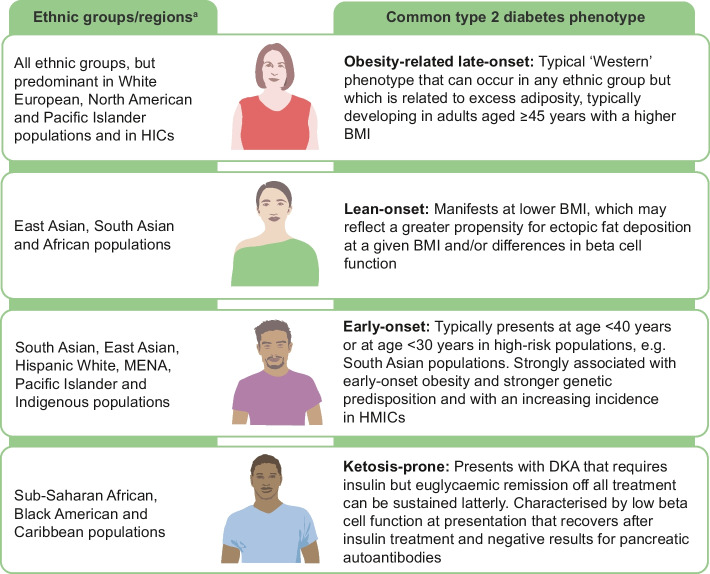
Schematic diagram illustrating common sub-phenotypes of type 2 diabetes and which ethnic groups they predominate in. ^a^All phenotypes can present in all ethnic groups but predominate in certain ethnic groups. HICs, high-income countries; HMICs, high- and middle-income countries. This figure is available as part of a downloadable slideset

Type 2 diabetes is not a uniform disease but encompasses multiple sub-phenotypes (Fig. 2) with distinct clinical features. While we recognise these sub-phenotypes in a clinical setting [2], and particular sub-phenotypes are often observed more frequently in particular ethnic groups [3], the treatment guidelines are the same, irrespective of sub-phenotype.

Early-onset type 2 diabetes (diagnosis at age <40 years) disproportionately affects non-European ethnicities but is on the rise in all ethnic groups [6]. Earlier presentation is associated with a significantly reduced life expectancy [46, 47], a higher risk of both microvascular [48] and macrovascular [46, 49] complications and worse pregnancy outcomes [50, 51]. Early-onset type 2 diabetes is typically characterised by severe obesity and the accumulation of adverse metabolic risk factors at presentation and appears to be common in South Asian populations, where earlier age at presentation is associated with diminished beta cell function [8, 40]. A Ugandan study showed that individuals with early-onset type 2 diabetes also had lower beta cell function [52].

Lean-onset type 2 diabetes, frequently observed in South and East Asian populations, is characterised by a lower BMI at diagnosis and relative insulin deficiency rather than insulin resistance [53, 54]. Notably, in the original UKPDS study, approximately one-third of participants had a BMI of <25 kg/m^2^ at diagnosis, challenging the common perception of type 2 diabetes as a disease of obesity alone [55]. A comparative cross-sectional study of BMI in type 2 diabetes between Indian individuals from the CARRS-Chennai study and non-Hispanic White individuals from the US NHANES survey demonstrated significant differences in risk at lean BMI; type 2 diabetes prevalence was 23.5% and 6.1% in Indian and White men, respectively, with a normal BMI (18.5–24.9 kg/m^2^), and 13.6% and 2.8% in Indian and White women, respectively, with a normal BMI [56]. Lean-onset type 2 diabetes and its consequences are poorly studied. Cross-sectional analyses suggest that lean-onset type 2 diabetes is associated with the need for earlier insulin treatment [57, 58]; however, large-scale epidemiological studies are hindered by the potential for misclassification, as adult-onset type 1 diabetes, pancreatic type 3C diabetes and other subtypes of diabetes can also present in adults with a lean BMI. Despite the substantial global prevalence of lean-onset type 2 diabetes, treatment algorithms remain predominantly focused on weight management, targeting the major obesity-driven type 2 diabetes phenotype in European ancestry groups.

In African and Caribbean populations, ketosis-prone type 2 diabetes represents another distinct phenotype. First described in the Flatbush region of New York [59], this form presents with diabetic ketoacidosis (DKA) at diagnosis but often transitions to insulin independence after initial treatment [60, 61]. This subtype has been increasingly recognised across sub-Saharan Africa but has also been observed in nearly all ethnic groups. As well as unprovoked DKA at presentation, ketosis-prone type 2 diabetes is characterised by sustained C-peptide production and negative results for pancreatic autoantibodies [62, 63]. The initial presentation is identical to that of type 1 diabetes cases that present with ketoacidosis and needs to be insulin-treated [61]. Subsequently, at follow-up, when antibodies are found to be negative and when there are reducing insulin requirements and sustained euglycaemic remission, a diagnosis of ketosis-prone type 2 diabetes should be considered [64].

The heterogeneity of type 2 diabetes across ethnic groups highlights the potential of precision medicine approaches that move beyond one-size-fits-all strategies. Importantly, though, variation also exists within ethnic groups; for example, although young-onset or lean-onset type 2 diabetes may be more prevalent among South Asian individuals, obesity-related late-onset type 2 diabetes is also commonly observed.

## Ethnic variation in type 1 diabetes

Type 1 diabetes exhibits substantial heterogeneity in its clinical presentation and underlying pathophysiology across different ethnic groups, although these differences are perhaps less well studied than in type 2 diabetes. Historically, the incidence of type 1 diabetes has been higher in White European populations than in other ethnic groups, with a prevalence of ~4 in 4000 [65]. However, crucially, this trend seems to be changing, as the projected rises in incidence are significantly higher in non-White European populations [65]. For example, in China, although the incidence is still low, it increased significantly from 2007 to 2017 (from 2.72 [95% CI 2.51, 2.93] to 3.38 [3.38, 3.78] per 100,000 person-years) and, in particular, the rise was notable in older age groups aged >30 years [66].

It is unclear if the observed ethnic variation in clinical characteristics of type 1 diabetes reflects biological differences or other social determinants of health. For example, in US-based studies, children from non-Hispanic Black and Hispanic backgrounds tend to present with more advanced disease at diagnosis, including a higher BMI, higher HbA_1c_ levels, more frequent DKA and a reduced likelihood of entering a partial remission phase, than non-Hispanic White children [67–69]. Children from non-European immigrant backgrounds in the Netherlands have also been found to present with more severe DKA at diagnosis than their Dutch counterparts, a disparity not fully explained by socioeconomic status [70].

A study analysing data from the Type 1 Diabetes TrialNet Pathway to Prevention Study found that Hispanic individuals had a lower risk of progressing from single to multiple autoantibody positivity than non-Hispanic White individuals. However, among children aged <12 years with multiple autoantibodies, being overweight or obese significantly increased the risk of developing type 1 diabetes, with the effect being more pronounced in Hispanic children [71]. Whether these differences reflect environmental or genetic/immune factors is not clear. A number of studies have shown lower rates of antibody positivity in non-European populations. In Thailand, a significant proportion of individuals with type 1 diabetes are autoantibody negative, and these individuals tend to be older at diagnosis and have a higher BMI and higher C-peptide levels, suggesting a different pathophysiological mechanism compared with that in autoantibody-positive individuals [72]. Similarly, several studies in sub-Saharan Africa have shown low rates of antibody positivity in phenotypically type 1 diabetes individuals, despite evidence of high genetic risk for type 1 diabetes [73, 74].

Genetic predisposition to type 1 diabetes does also show variability across ethnicities. High-risk HLA haplotypes, such as *HLA-DR3*/*DR4* and *HLA-DQ8*, are more prevalent in European populations and less common in some Asian groups, potentially influencing disease susceptibility and presentation [73, 74]. Internationally, distinct subtypes such as fulminant type 1 diabetes have been reported in East and South Asian populations, characterised by abrupt onset, negative autoantibodies and intense immune infiltration of the pancreas, and often linked to specific HLA alleles (e.g. DRB1*04:05-DQB1*04:01) [75–77].

Although less well studied than type 2 diabetes, these findings highlight the importance of considering ethnic-specific factors in type 1 diabetes.

## The impact of lack of ethnic diversity in precision diabetes medicine

Three case studies are presented in the text box that collectively illustrate how the lack of ethnic and ancestral diversity in discovery, validation and guideline development has led to persistent diagnostic and therapeutic blind spots. The recent re-emergence of malnutrition-related diabetes mellitus (MRDM) as a distinct entity also highlights the issue perfectly. For many years there has been considerable debate regarding unusual presentations of diabetes in LMICs and/or tropical regions [78]. These phenotypes include MRDM (previously described as J-type diabetes or protein-deficient pancreatic diabetes) and fibrocalcific pancreatic diabetes. MRDM was declassified by the WHO in 1999 because of a lack of definitive evidence linking malnutrition directly to diabetes [79]. Studies now demonstrate that individuals with MRDM (typically with a low BMI, preserved insulin sensitivity and impaired insulin secretion) cannot be adequately classified under existing categories [80]. Its prevalence remains uncertain, partly because it disproportionately affects populations with limited access to diagnostic resources and research infrastructure. Fibrocalcific pancreatic diabetes is associated with pancreatic calcification and presents with both diabetes and exocrine failure, typically in tropical regions and in people who are lean [78, 81]. In both cases, lack of research has led to confusion around nomenclature, the validity of the subclassification itself (with some believing these presentations to be sub-phenotypes of type 1 or type 2 diabetes [82]) and, critically, optimal management. Addressing this gap requires renewed investment in research within LMICs to refine diagnostic criteria and elucidate underlying mechanisms, enabling appropriate classification and management of these overlooked forms of diabetes.
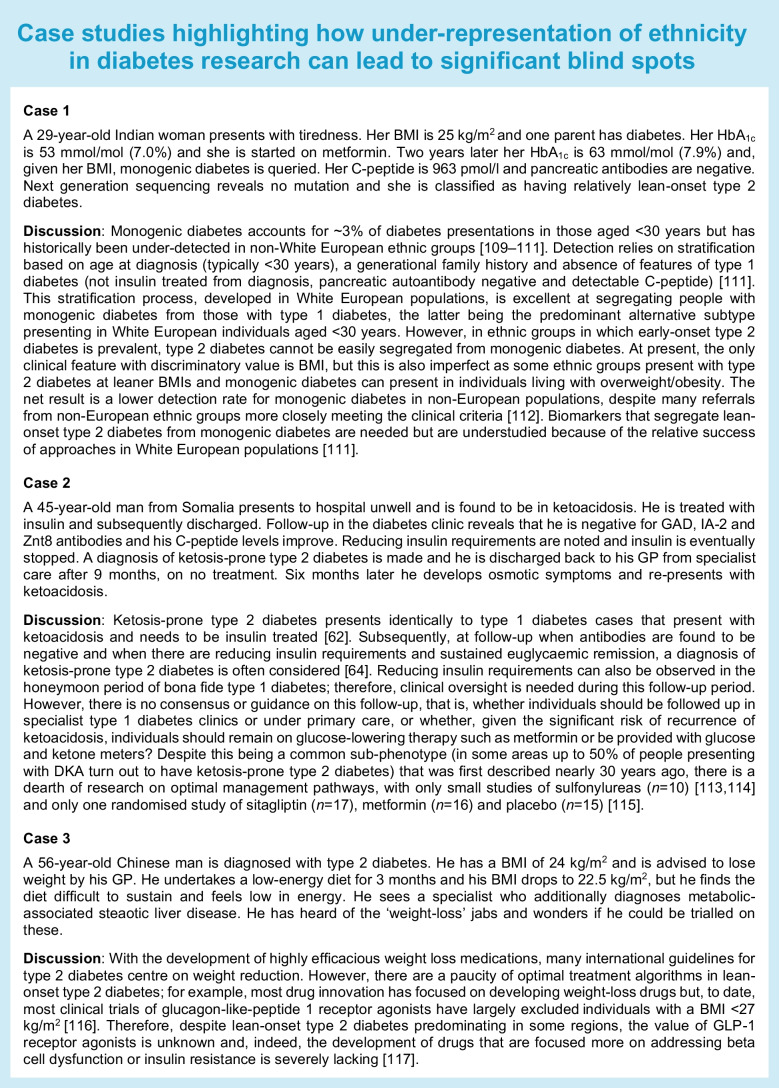


## Incorporating ethnicity into precision medicine for diabetes

To translate the observed ethnic differences in the presentation, progression and complications of diabetes into precision medicine solutions, critical questions must be raised. Should ethnicity as a categorisation guide distinct approaches to care, or can its effects be captured more effectively through individualised markers, for example phenotypic traits or polygenic scores?

Clinically, the ability to stratify individuals by risk in order to escalate interventions for those most vulnerable and de-escalate interventions for those at lower risk is central to precision medicine. Ethnicity can act as a proxy for underlying differences in genetic risk, pathophysiology, environmental exposures and access to care. However, uncritical or indiscriminate use of ethnic classifications risks reinforcing inequities rather than addressing them. Over-reliance on ethnicity may further obscure the more proximal causes of disparities, such as structural determinants and individual-level risk factors, that have nothing to do with genetic ancestry [17, 83].

If equity is assumed to be achievable, the next consideration is whether ethnicity-informed approaches demonstrably outperform conventional, one-size-fits-all models; at present, data to support this notion are severely lacking. While there are theoretical and empirical arguments in favour of tailoring approaches, the challenge lies in how ethnicity is ‘operationalised’ for precision medicine. At one end of the spectrum there are approaches anchored in genotypes and or genotype–phenotype associations [9, 30, 31, 39]; these at least seem unbiased but if the outcome measurements themselves are influenced by social, environmental and systemic factors then even genetically anchored approaches risk bias. At the other end of the spectrum, an approach based on ethnicity as a proxy for biological variation is likely to be reductionist, failing to capture within-group variation and influences from social determinants of health.

Furthermore, how granularly should we capture ethnicity or ancestry and how do we account for population admixture that may lead to hybrid phenotypes? The answers are not clear. Meanwhile, the widespread practice of aggregating individuals into broad ethnic categories, such as ‘African’ or ‘Asian’, fails to reflect the extensive heterogeneity within these populations and risks concealing important differences in disease risk and treatment response [84]. These issues are not easy to solve, but a critical first step is for researchers and clinicians to consider carefully and reflect on how their precision medicine solution works in diverse ancestries.

## Conceptual models

**Fig. 3:**
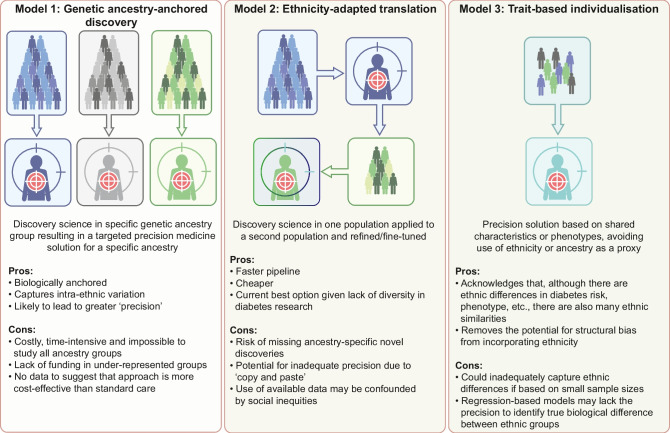
Conceptual models for how ethnicity could be incorporated into precision medicine tools. The pros and cons of each model are shown. These models represent complementary rather than mutually exclusive strategies. This figure is available as part of a downloadable slideset

Broadly, three conceptual models can be considered for incorporating ethnicity into precision diabetes medicine (Fig. 3).

### Genetic ancestry-anchored discovery

Genetic ancestry-anchored discovery entails conducting discovery studies within specific genetic ancestry populations. This model provides the strongest scientific foundation for uncovering true biological heterogeneity, especially genotype-driven approaches, which serve as a stable marker of biological risk, independent of environmental and social context, allowing for the identification of aetiological heterogeneity that is less prone to confounding [85]. A good example is the identification of a variant in *G6PD* (Val98Met [rs1050828]) [86], which lowers HbA_1c_ levels in African individuals to such an extent that it may lead to underdiagnosis of diabetes if based solely on HbA_1c_. This discovery was made through a trans-ancestry genome-wide association study (GWAS) but, even then, the diversity of those included in the study was poor. Another example is the development of polygenic scores for type 1 diabetes, which have shown utility in predicting those at high risk of type 1 diabetes [87], an important step for the future as efficacious immune-modulating agents are developed and become widely available [88]. These scores have shown some portability to other populations, for example a score derived in White populations showed good performance in Asian Indian populations [89]. However, a GWAS in an African population identified African-specific HLA haplotypes in addition to European haplotypes that performed significantly better in predicting type 1 diabetes than the European genetic risk score (AUC 0.871 vs 0.798, respectively) [90].

The scalability of ancestry-anchored discovery is limited by cost, sample availability and the complexity of recruiting adequately powered cohorts across *all* global ancestries.

### Ethnicity-adapted translation

The second strategy, ethnicity-adapted translation, involves applying findings derived in one population, typically of European ethnicity (or ancestry for genetic approaches), to other ethnic groups through post hoc adjustments. This approach has been widely adopted in the context of polygenic scores for diabetes, with European-derived scores re-evaluated in other populations to assess transferability and performance [91]. While this method is more practical and resource efficient, it may fail to capture ancestry-specific variants and interaction effects, leading to reduced performance in under-represented groups (as outlined in the above African example). Polygenic scores derived from multi-ancestry GWAS appear to capture a larger fraction of phenotypic variation in a given ancestry group, even if it includes variants that were not associated with the disease at genome-wide significance level in all ancestries [10]. Another example of ethnicity-adapted translation is the replication of data-driven diabetes clusters derived from a Scandinavian population using HbA_1c_, age at diagnosis, GAD antibodies, BMI, HOMA-B and HOMA-IR [92]. Crucially, these clusters have different trajectories in terms of progression to needing insulin, the development of complications and, indeed, mortality. This approach has been rapidly replicated in a number of ethnicity groups, for example Chinese and Hispanic populations [93]. However, some studies have undertaken de novo clustering rather than replication of the original clusters. For example, an Indian study of 19,000 individuals with type 2 diabetes found novel clusters of type 2 diabetes that were phenotypically distinct from the original Scandinavian clusters, and two of the Scandinavian clusters could not be directly replicated [94].

Altogether, these examples illustrate how precision medicine findings in one population can be both transposed and expanded on in others, yielding novel insights. However, it also highlights the necessity of rigorous external validation and illustrates that direct transposition across populations is rarely sufficient.

### Trait-based individualisation

The third model, trait-based individualisation, avoids using ethnicity or ancestry as a proxy altogether and instead stratifies individuals based on directly measurable characteristics such as BMI, HbA_1c_ or other biomarkers. This approach underlies emerging subtype frameworks in type 2 diabetes, in which individuals are grouped by clinical or molecular phenotypes rather than by race or ethnicity. Although more equitable and clinically actionable, this model may underperform in populations in which relevant ethnicity-linked features are not well captured by standard biomarkers. A recent example of this type of approach is a data-driven model (derived from UK population data) for predicting the best glucose-lowering therapy to achieve HbA_1c_ reduction that used nine clinical variables cross-sectionally: age, duration of diabetes, sex, baseline HbA_1c_, BMI, eGFR and HDL-cholesterol, total cholesterol and alanine aminotransferase levels [95]. Use of the model achieved better HbA_1c_ reduction at 12 months than real-world standard care. Ethnicity as a variable was not incorporated into the model, but an analysis stratified by self-reported ethnic group showed no differences in performance across groups. The analysis comprised data from only ~15,000 South Asian and ~7000 Black individuals and so needs to be replicated in other datasets, but this model shows promise.

## Reconciling conceptual models for equitable precision medicine

The conceptual models presented in this review represent complementary, rather than mutually exclusive, strategies. A reconciled framework that strategically leverages their respective strengths offers the most pragmatic and equitable route towards implementation. However, to advance to real-world impact, the field must also consider additional dimensions, including ethnicity (or regional)-specific environmental exposures [96], life-course influences [97] and implementation science, when applying these models across diverse populations.

A translational roadmap to clinical implementation might comprise the following four stages:

1. Expand existing and develop novel methods for discovery science: Increased investment in genomic, transcriptomic and phenotypic datasets from ancestrally diverse populations is urgently needed. This can be achieved through multi-ethnic biobanks, regional disease registries and locally led cohort studies. Traditional prospective cohorts are often prohibitively expensive and time-consuming and a more sustainable model may involve embedding discovery efforts into routine healthcare delivery, through structured electronic health records, linked biosample collections and opportunistic case finding within clinical workflows. Approaches such as federated data analysis, digital phenotyping and public–private partnerships could help reduce costs and enhance scalability, particularly in LMICs [98, 99].
2. Validation and adaptation of existing tools: Precision tools, such as polygenic risk scores, machine-learning algorithms and clustering frameworks, must be systematically re-evaluated in ethnically diverse populations. This includes recalibration of parameters, potential incorporation of other relevant parameters and external validation in local datasets and testing across ancestral subgroups. A key step in this process will be the establishment of consensus standards for how ethnicity and ancestry are reported, including consensus on the use of self-reported vs genetically inferred categories, level of granularity (e.g. disaggregated vs pan-ethnic classifications) and how these variables are integrated into predictive models. Transparent and consistent reporting will be essential to ensure rigour and comparability across studies.
3. Develop scalable, trait-based clinical algorithms: Algorithms based on routinely collected variables can be rapidly implemented, especially in low-resource settings. These approaches offer a low-cost entry point to precision medicine, particularly when genomic infrastructure is limited. Nonetheless, trait-based models must be locally validated and periodically updated to reflect demographic and epidemiological shifts.
4. Integrate equity into implementation science: Without explicit attention to equity, precision medicine risks entrenching existing structural disparities. Implementation frameworks must be adapted to available healthcare resource, cultural relevance and sociopolitical contexts [99]. Community engagement in the co-design of interventions is essential to foster trust, improve uptake and ensure relevance. Additionally, models should consider life-course and environmental exposures (e.g. urbanisation, nutrition, infection, stress), which often vary by ethnicity and interact with genetic susceptibility to influence diabetes risk and progression [97, 100].

A key priority for the field will be to establish consensus frameworks for how ethnicity is defined, measured and operationalised in precision medicine research, and to embed ancestral and ethnic diversity at every stage, from discovery science to implementation and evaluation.

## Barriers, solutions and research gaps

Despite growing momentum in precision diabetes medicine, several systemic, structural and scientific barriers continue to hinder the equitable application of these approaches in ethnically diverse and low-resource settings (Table 1). While precision medicine holds promise for advancing equity, many disparities in diabetes care stem from entrenched structural and social determinants. Arguably, a more inclusive application of evidence-based medicine, addressing barriers such as health literacy, access to care and underdiagnosis, could close equity gaps even without precision tools. Practical measures such as culturally adapted care, broader diagnostic reach for under-represented groups and locally tailored interventions remain essential. Precision strategies should build on these foundations rather than replace them. An important open question is whether targeted improvements within existing care models might, in some settings, provide more immediate or scalable equity gains than precision approaches. These need not be mutually exclusive; pursuing both in parallel may provide the greatest benefit.

**Table 1:** Barriers to and solutions for achieving better ethnic diversity in precision medicine for diabetes and current research gaps

Category	Barrier	Solution	Research gaps
Lack of diversity in discovery science	A fundamental limitation is the lack of representation of non-European populations in genomic and clinical research. In recent estimates, >90% of GWAS have been conducted in individuals of European ancestry, despite the global burden of type 2 diabetes being highest in the Global South [101].	Dedicated investment in ancestry-anchored discovery studies and multi-ethnic biobanks is urgently needed. Ongoing efforts such as All of Us in the USA [103] and Our Future Health (20% non-White participants) in the UK (https://ourfuturehealth.org.uk/) are steps in the right direction, but global initiatives must also prioritise under-represented regions and populations [102]. Country-specific disease registries such as the Hong Kong Diabetes Registry have shown the power of routinely collected data and bio-banked samples in driving discovery and validation [103].	• How can we expand cost-effective ancestry-anchored genomic and phenotypic discovery efforts to under-represented populations? • What actionable biological insights might be uncovered through multi-ethnic, ancestry-specific diabetes research? • How can global consortia be structured to ensure equitable contributions and leadership from LMIC-based researchers? • What tools can help disentangle within-group heterogeneity (e.g. among ‘South Asian’ or ‘African’ populations) to avoid masking important biological and social variation? • How can we integrate multi-dimensional data (genomics, exposomics, sociocultural) to better reflect intra-ethnic diversity?
Incomplete and inconsistent recording of ethnicity	In many healthcare systems, ethnicity data are missing, inconsistently recorded or misclassified. This limits the ability to evaluate disparities, tailor interventions and conduct ancestry-aware analyses. Moreover, ethnicity is often captured as self-reported categories that do not align with genetic ancestry or reflect meaningful cultural or environmental exposures. Ethnicity labels are often shaped by regulatory or national frameworks rather than scientific rationale (e.g. US Food and Drug Administration-mandated census categories vs France’s policy of not collecting ethnicity data), which can obscure meaningful biological or sociocultural variation relevant to precision medicine.	Standardisation of ethnicity and ancestry recording across health systems and research platforms is essential [104] for example, in the USA, ‘Asian’ typically refers to East Asian ancestry, whereas in the UK it refers to South Asian ancestry. Real-world data has huge potential to derive precision medicine insights but the aggregated and inconsistent description of ethnicity prevents meaningful insights.	• What are the most reliable and standardised ways to capture ethnicity and genetic ancestry in clinical and research datasets? • How do we reconcile self-identified ethnicity with genomic ancestry in a way that is scientifically robust and socially responsible? • Can international consensus be achieved on nomenclature and groupings?
Underpowered subgroup analyses in clinical trials	Non-European ethnic groups are frequently under-enrolled in RCTs of diabetes treatments [105]. Even when enrolled, sample sizes are often too small to allow meaningful subgroup analysis. As a result, studies often only report analyses for White European ethnicity.	The provision of ethnic group data in supplementary files could permit subsequent meta-analysis to boost power. When RCTs are not feasible, real-world evidence from diverse routine care datasets can be leveraged, but coding of ethnicity is often poor / inconsistent.	• How can future clinical trials be designed to ensure adequate statistical power for meaningful ethnicity-stratified analysis? • What strategies will enable the pooling and meta-analysis of under-reported subgroup data across existing trials?
Technological and resource constraints	High-precision tools, such as polygenic risk scores, multi-omics assays and digital decision support systems, may not be widely available in LMICs or underserved areas, raising concerns about scalability and global equity. Even simple tools such as C-peptide testing remain underused in many settings.	However, as outlined, precision medicine need not be high tech to be high impact. Stratification based on routinely collected clinical variables (e.g. BMI, HbA_1c_, age at onset) can be implemented at scale using low-cost decision support tools. Whether precision medicine for diabetes can be scaled across all healthcare settings remains uncertain. Even established tools such as monogenic diabetes testing are not universally accessible. Critics often point to the high perceived costs and argue that greater population-level impact could be achieved by improving access to existing, simpler care models [106]. Moreover, while precision approaches may provide long-term savings through better diagnosis and treatment targeting, these benefits are difficult to quantify in the short term [107]. Investments in point-of-care diagnostics and mobile health technologies can also extend reach in low-resource settings. The case for precision medicine in low-income settings is reviewed in detail elsewhere [99].	• What low-cost, scalable precision tools (e.g. C-peptide-based stratifiers or simplified decision algorithms) can be validated for use in low-resource settings? • Can point-of-care diagnostics or digital health platforms be leveraged to deliver precision care in LMICs effectively and equitably? • How can we make better use of real-world data to ascertain these insights? • What is the real-world clinical utility of precision medicine tools (e.g. subtype-based algorithms, polygenic scores) in improving outcomes across diverse populations? • Can we quantify the cost-effectiveness of implementing precision approaches vs current standard care, particularly in LMICs or underserved settings?
Risk of exacerbating health inequities	If precision medicine tools are developed and deployed without attention to structural inequities, they risk worsening existing disparities. This includes unequal access to testing, algorithms that encode systemic bias, and the potential for misclassification when applying population-derived tools to individuals from different backgrounds.	To prevent worsening of existing disparities, equity must be embedded at every stage of precision medicine development, from hypothesis generation to implementation. Implementation is a key issue in driving inequalities, as lack of accessibility due to cost may become an issue. However, it is important to note that precision medicine solutions can also address health inequalities. In a study of real-world data, the OR for misclassification of type 1 diabetes was approximately threefold higher in non-White individuals than in self-reported White individuals and clinical prediction models performed poorly. Inclusion of a type 1 diabetes polygenic score in the algorithm improved the accuracy of type 1 diabetes prediction, mitigating potential clinical biases in diagnosis [108].	• How can precision medicine solutions be designed to reduce, rather than reinforce, structural biases in diabetes diagnosis and care? • What implementation models ensure equitable access to precision tools across socioeconomic and ethnic groups? • What safeguards are needed to avoid algorithmic bias when deploying risk scores or prediction models derived from majority populations? • How can we involve minoritised communities in the design, dissemination and implementation of precision medicine solutions? • What are the barriers to trust and engagement in genomics research among ethnic minority groups and how can they be addressed? • Is a precision medicine approach superior to standard care?

## Conclusions

Precision medicine offers a valuable opportunity to tailor diabetes care to individual and population-level differences, but its potential will only be realised if it is equitable. Ethnic variation in diabetes risk, phenotypes and treatment response is well established, yet remains under-represented in the evidence base informing precision approaches. Applying tools derived primarily in White European populations without adequate validation risks reduced performance and may inadvertently perpetuate disparities.

Rather than discarding ethnicity as a variable, we must use it thoughtfully, acknowledging both its limitations and its potential value as a proxy for underlying biological and sociocultural factors. Progress will require greater diversity in discovery science, improved recording and standardised use of ancestry and ethnicity data, and investment in scalable models that work across healthcare settings.

Moving forward, the focus must be on building precision medicine frameworks that are robust, generalisable and inclusive, ensuring that advances in diagnosis and treatment deliver meaningful benefits to communities affected by diabetes.

## Supplementary Information

Below is the link to the electronic supplementary material.

Slideset of figures (PPTX 705 KB)

## Abbreviations

DKA: Diabetic ketoacidosis
GWAS: Genome-wide association study
LMIC: Low- and middle-income countries
MENA: Middle Eastern and North African
MRDM: Malnutrition-related diabetes mellitus
