# Supplementary material for: Ethnic diversity in precision medicine: a reality or an aspiration?
Source: Diabetologia. 2025 Aug 7;68(11):2449–64. doi: 10.1007/s00125-025-06513-4 (PMC12534348; doi:10.1007/s00125-025-06513-4)
Supplement: Supplementary file 1 — Slideset of figures (PPTX 705 KB) [file 125_2025_6513_MOESM1_ESM.pptx]

## Slide 1
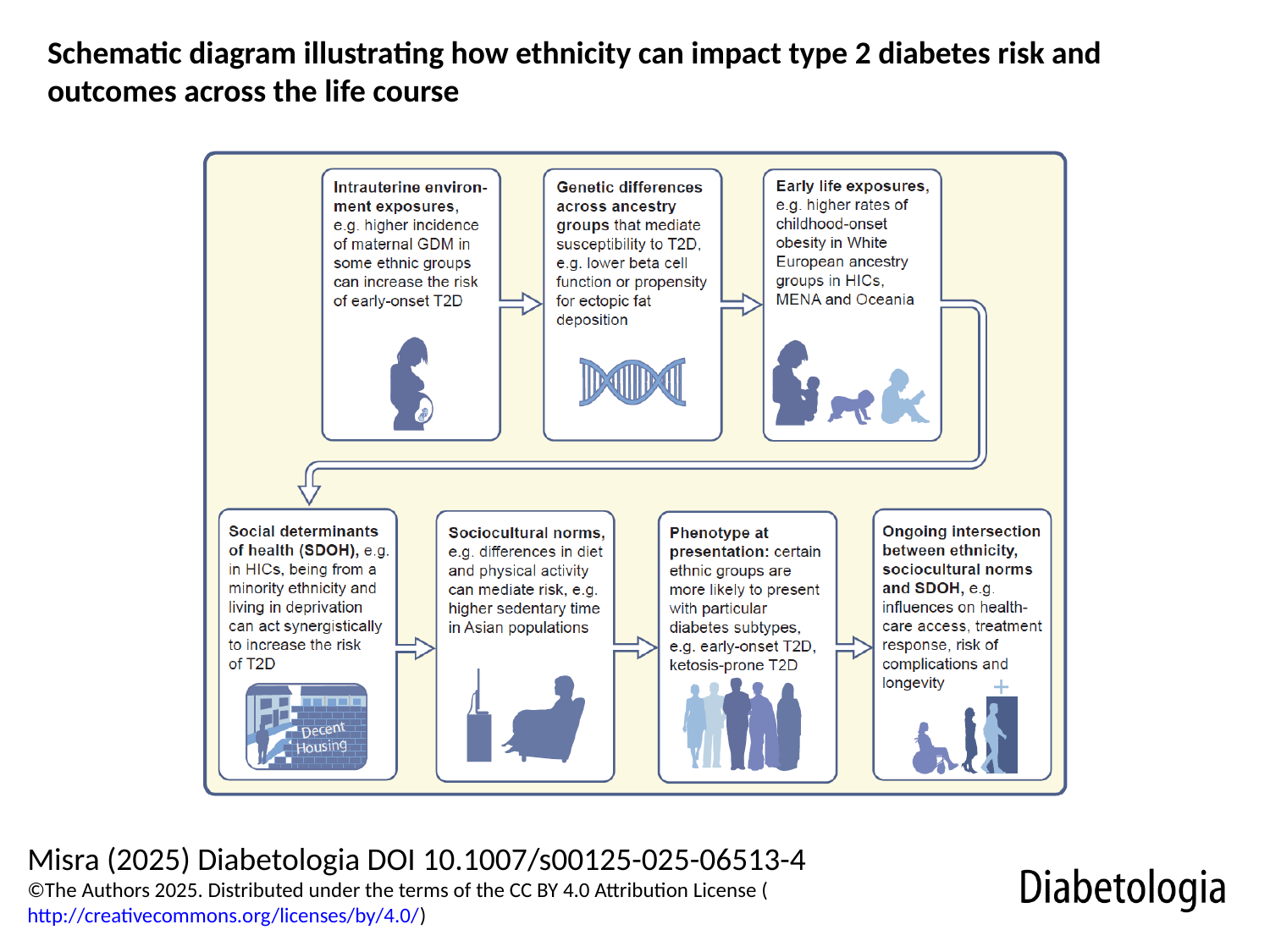

Schematic diagram illustrating how ethnicity can impact type 2 diabetes risk and outcomes across the life course
Misra (2025) Diabetologia DOI 10.1007/s00125-025-06513-4
©The Authors 2025. Distributed under the terms of the CC BY 4.0 Attribution License (http://creativecommons.org/licenses/by/4.0/)

## Slide 2
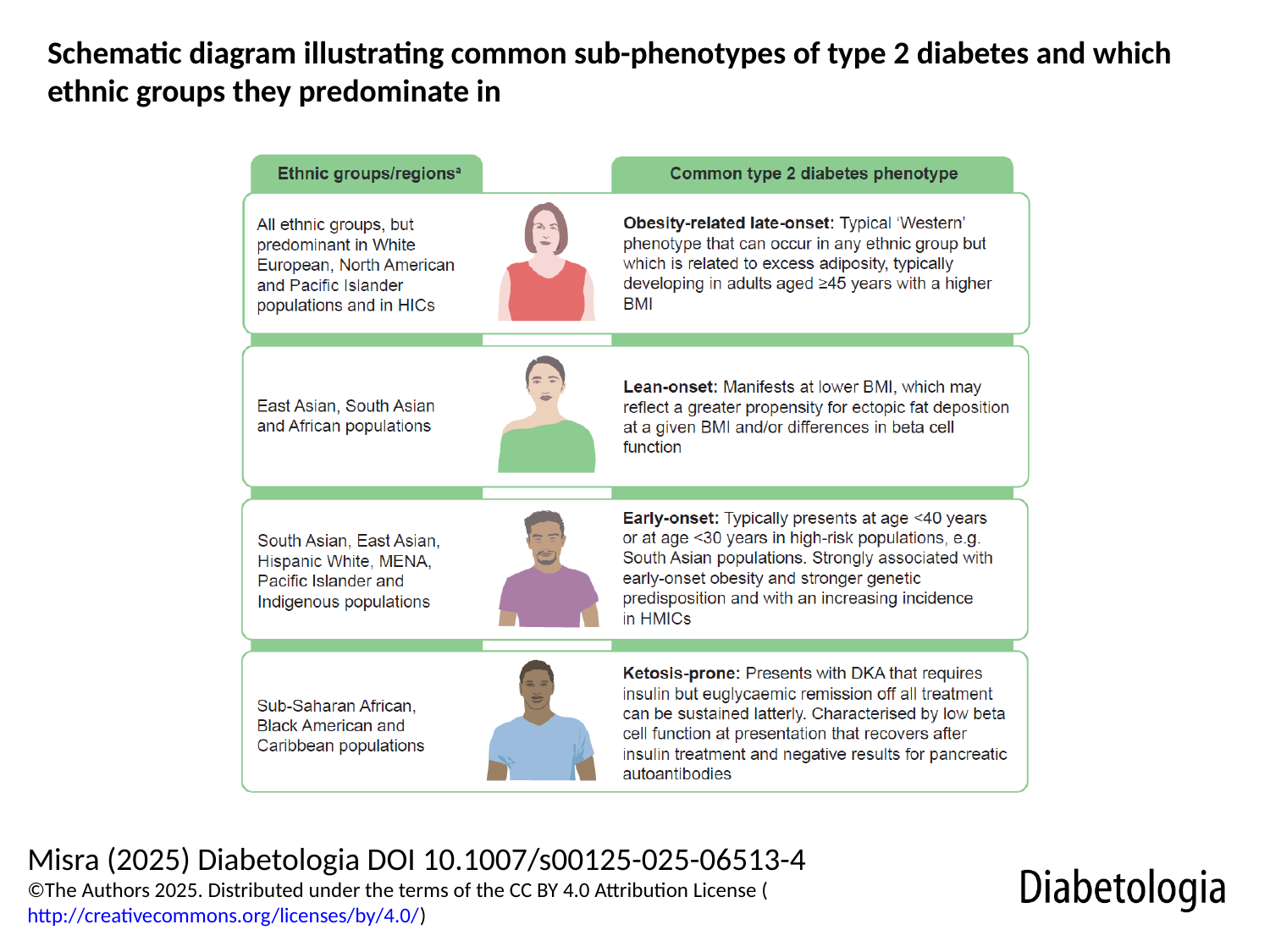

Schematic diagram illustrating common sub-phenotypes of type 2 diabetes and which ethnic groups they predominate in
Misra (2025) Diabetologia DOI 10.1007/s00125-025-06513-4
©The Authors 2025. Distributed under the terms of the CC BY 4.0 Attribution License (http://creativecommons.org/licenses/by/4.0/)

## Slide 3
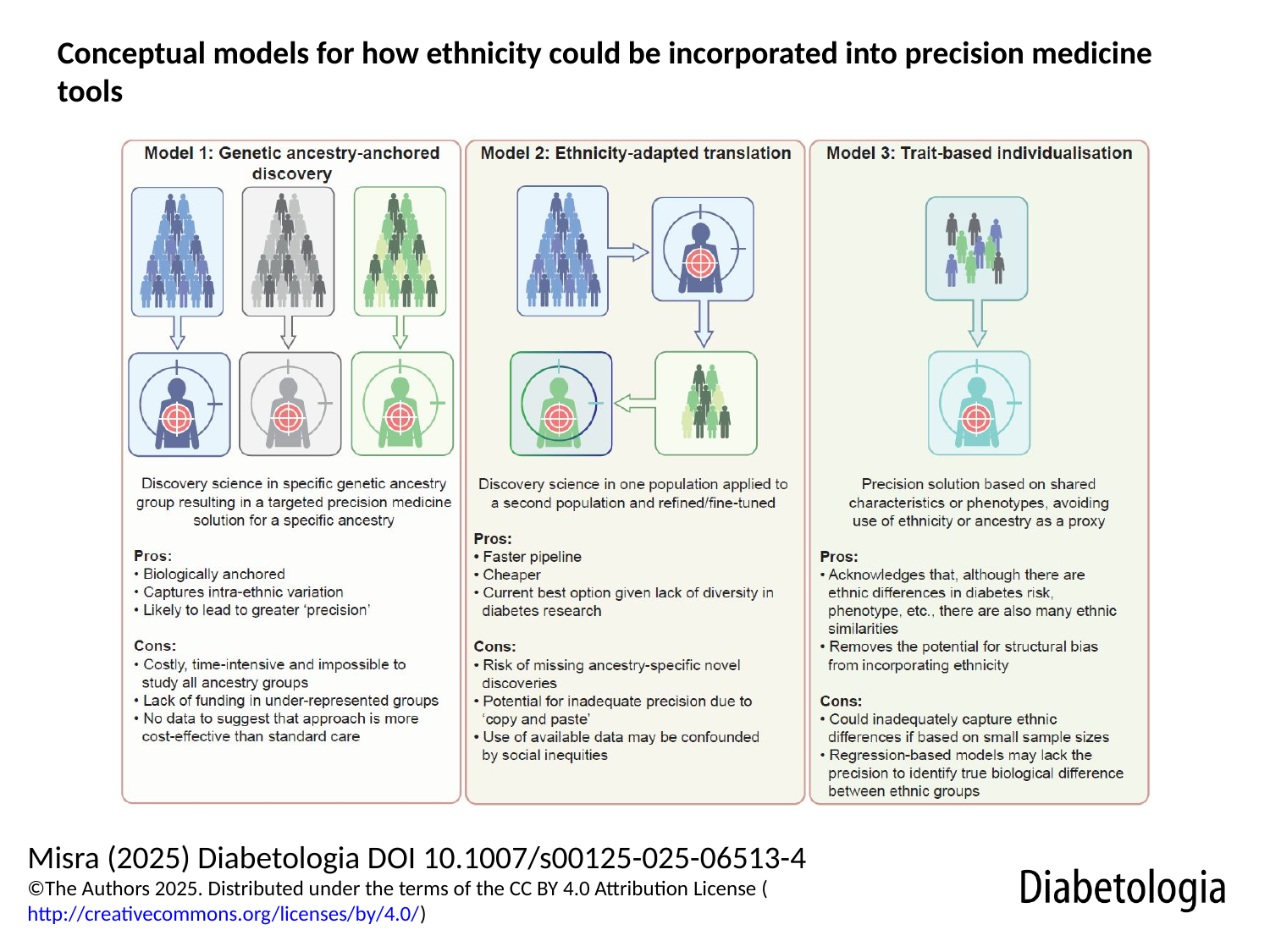

Conceptual models for how ethnicity could be incorporated into precision medicine tools
Misra (2025) Diabetologia DOI 10.1007/s00125-025-06513-4
©The Authors 2025. Distributed under the terms of the CC BY 4.0 Attribution License (http://creativecommons.org/licenses/by/4.0/)
